# Sequenced Breakpoints of Crossover Suppressor/Inversion *qC1*

**DOI:** 10.17912/micropub.biology.000494

**Published:** 2021-11-03

**Authors:** Mark L. Edgley, Stephane Flibotte, Donald G Moerman

**Affiliations:** 1 Department of Zoology, University of British Columbia, Vancouver, British Columbia, Canada; 2 UBC/LSI Bioinformatics Facility, University of British Columbia, Vancouver, British Columbia, Canada

## Abstract

We used whole-genome sequencing (WGS) data from a number of balanced lethal strains in *Caenorhabditis elegans* to show that the crossover suppressor *qC1* is an inversion. The rearrangement is complex, with a large primary inversion that contains several other smaller inverted regions. The graphical representation below depicts these various *qC1 *rearrangements for ease of conceptualization. It is the simplest chromosomal structure compatible with the data currently available, but even then it is worth noting that the complexity of the *qC1* chromosome can make the graphical reconstruction difficult to understand, and it may seem a bit like relativity theory or artwork from M.C. Escher (https://moa.byu.edu/m-c-eschers-relativity/).

**Figure 1. Chromosome III in the qC1 balancer:**
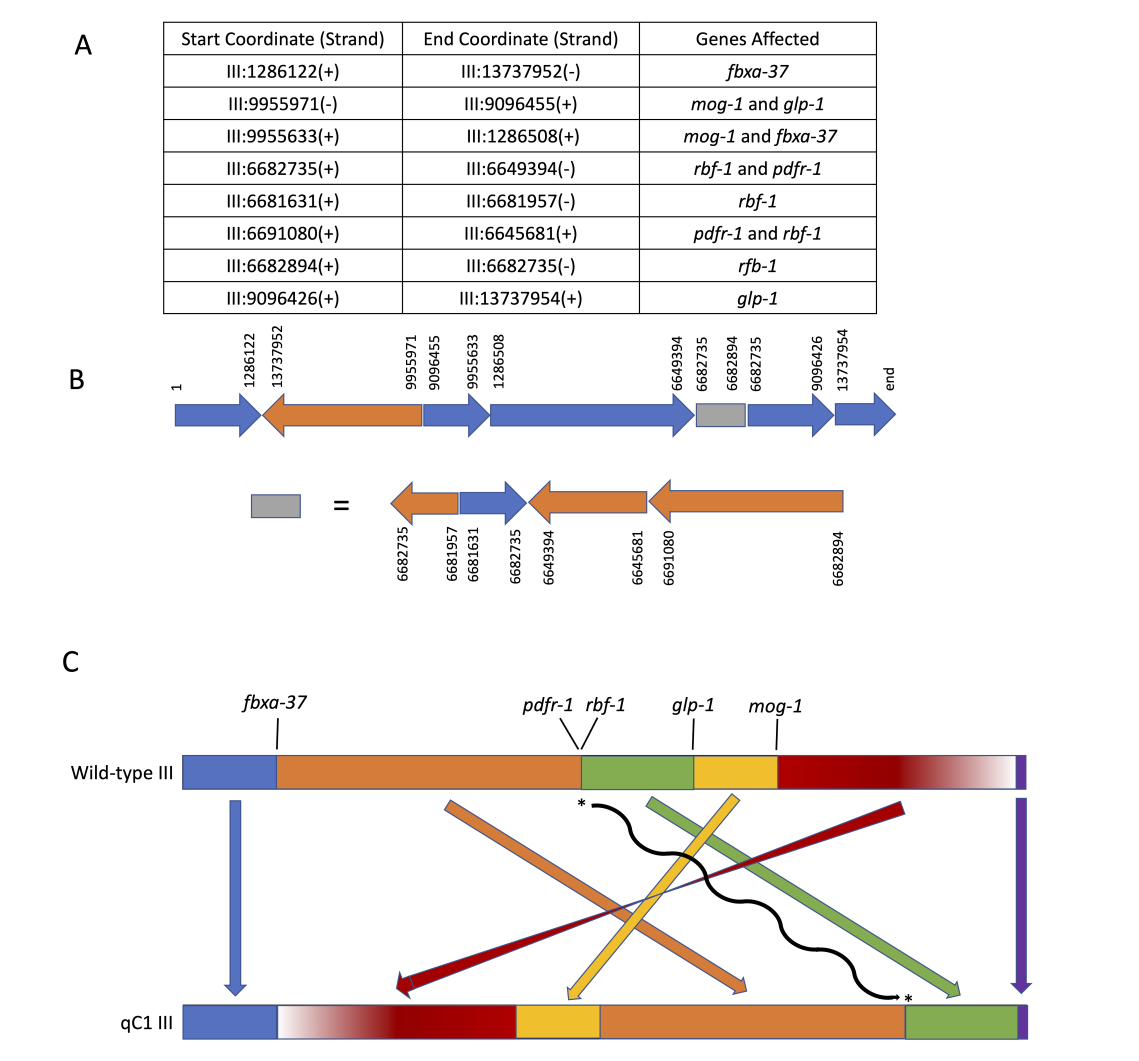
(**A**) Breakpoint connections deduced by aligning split reads at the breakpoints. Identical strands indicates that the sequence on the left is connected directly to the sequence on the right, while different strands denotes a change of strand (inversion). Genes affected by the breakpoints are listed in the third column. (**B**) A schematic representation of chromosome III deduced for *qC1* is shown. Each arrow represents the chromosomal segment of the reference sequence with the corresponding coordinates listed. The direction and color of each arrow represent the strand/direction of the sequence within the original reference chromosome. For simplicity of visualization, the complex rearrangement around 6.6Mb is shown separately. (**C**) A further simplified schematic representation of the chromosome rearrangements in qC1. Only the genomic segment represented in red is inverted relative to the wild-type chromosome III. Genes at breakpoints are indicated and the asterisks linked with the black wavy arrow represent the complex structural variation near 6.6Mb.

## Description

Classical dominant intrachromosomal crossover suppressors in *C. elegans* (e.g. *mnC1*, *sC1*, *qC1, mC6* [now *mIn1*]) have long been thought likely to be inversions. Prior to economical genome sequencing, proof of inversion depended on the isolation of crossover suppressor variants differentially marked with morphological mutations, construction of homozygous suppressor genotypes with different such markers *in trans*, and recombination analysis within and outside the putative inverted region (*hIn1*: Zetka and Rose, 1992; *mIn1*: Edgley and Riddle, 2001).

Here we report genome sequence data confirming that the crossover suppressor *qC1* (Austin and Kimble, 1989) is a complex inversion (**Fig. 1**). The data used in this analysis were generated in the course of genome sequencing to identify causative lesions in lethal mutations balanced by *qC1* (Li-Leger *et al.*, 2021).

The primary inverted region in *qC1* runs from LG III coordinates 1,286,123 through 13,737,951; these physical breakpoints correspond to the genetic limits of balancing behavior observed in recombination analysis of marker/*qC1trans* heterozygotes (*dpy-1* to *tra-1*, summarized in Edgley *et al.*, 1995). In addition to the primary inversion, the *qC1*chromosome is characterized by several regions exhibiting further rearrangement, possibly the result of the radiation dose used to generate the suppressor (7200 R gamma). The original balancer was known to carry lesions in *glp-1* and *mog-1* that were not present in the strain mutagenized to generate *qC1* and not characterized molecularly; these lesions were both identified as additional inversion breakpoints inside the primary *qC1* inversion. In particular, as can be seen in **Fig. 1**, *glp-1(q339)* consists of two breakpoints within the gene at chromosome coordinates 9,096,426 and 9,096,455, which in addition to breaking the gene deletes 28 base pairs from one of its coding exons. A similar event is seen in *mog-1*, with breakpoints at coordinates 9,955,633 and 9,955,971.

The schematic representation of *qC1* chromosome III shown in Fig. 1B and1C is the simplest structure compatible with the breakpoints and breakpoint connections identified in the current study. However, we cannot exclude the possibility that additional breakpoints have been undetected, which would result in more complex chromosomal rearrangements.

## Methods

**Sequence Generation**

Large asynchronous populations of balanced heterozygous strains were harvested by washing freshly-starved 100 mm standard agar/OP50 culture plates with M9 buffer. Worms were pelleted in 15 ml centrifuge tubes, the supernatant was removed by aspiration, and the packed worms were used to make purified DNAs using standard extraction protocols. These DNAs were subjected to sequencing on the Illumina platform and the data were subsequently analyzed in-house. The strains with qC1 balancer analyzed in the current study were sequenced at an average depth of coverage ranging from 43x to 48x (mean and median of 46x).

**Sequence Analysis**

Read alignments created for the study by Li-Leger *et al.*. (2021) were first visually inspected within genes known to be mutated in *qC1* (i.e. *glp-1* and *mog-1*) using the IGV genome viewer (Thorvaldsdóttir *et al.*., 2013) in order to locate obvious breakpoints in chromosome III. LUMPY (Layer *et al.*, 2014) was then used to search for potentially missed breakpoints within the whole genome. Candidate breakpoints were kept for further investigation only when they were present in strains containing the *qC1* chromosome and absent in strains without that balancer. Each breakpoint was then further analyzed by aligning split reads at the breakpoint using the blast tool available on the WormBase website (https://wormbase.org/tools/blast_blat), which allowed connecting two breakpoints to one another as reported in **Fig. 1A**. A mutated chromosome III for *qC1* was then deduced using those breakpoint connections and a parsimonious approach while taking into account the copy-number evidence provided by the read coverage in the regions of interest. All analysis was done using the reference genome in WormBase (https://wormbase.org, WS282).

## Reagents

DNA sequence analysis was based on the wild type N2 Bristol derived strain PD1074, the Million Mutation Project strain VC30189 and several *qC1* and *nT1* strains that were derived from a parental strainfollowing mutagenesis with EMS (ethyl methane-sulfonate). Each of the strains resulting from mutagenesis harboured a lethal mutation *in cis* with *unc-32* balanced by *qC1 or in cis* with *unc-24 balanced by nT1*. Further details for these strains can be obtained in Li-Leger *et al.*. (2021).

**Table:** 

**Strain**	**Genotype**	**Source**
PD1074	N2 derivative	CGC
GE2220	*unc-32(e189) top-3(t1516)/qC1[dpy-19(e1259) glp-1(q339)] III; him-3(e1147) IV*	Li-Leger *et al*., 2021
GE2348	*unc-32(e189) zyg-8(t1518)/qC1[dpy-19(e1259) glp-1(q339)] III; him-3(e1147) IV*	Li-Leger *et al*., 2021
GE2352	*unc-32(e189) cyk-3(t1535)/qC1[dpy-19(e1259) glp-1(q339)] III; him-3(e1147) IV*	Li-Leger *et al*., 2021
GE2357	*unc-32(e189) cls-2(t1527)/qC1[dpy-19(e1259) glp-1(q339)] III; him-3(e1147) IV*	Li-Leger *et al*., 2021
GE2367	*unc-32(e189) klp-19(t1563)/qC1[dpy-19(e1259) glp-1(q339)] III; him-3(e1147) IV*	Li-Leger *et al*., 2021
GE1958	*him-9(e1487) II; unc-24(e138) atg-7(t1726)/nT1[let(m435)] IV; dpy-11(e224)/nT1[let(m435)] V*	CGC
GE2391	*him-9(e1487) II; unc-24(e138) dif-1(t1932)/nT1[let(m435)] IV; dpy-11(e224)/nT1[let(m435)] V*	CGC
GE2881	*him-9(e1487) II; unc-24(e138) F56D5.2(t1744)/nT1[let(m435)] IV; dpy-11(e224)/nT1[let(m435)] V*	Li-Leger *et al*., 2021
GE2890	*him-9(e1487) II; unc-24(e138) C34D4.4(t1821)/nT1[let(m435)] IV; dpy-11(e224)/nT1[let(m435)] V*	Li-Leger *et al*., 2021
VC30189	Million Mutation Project strain. This strain was isolated after ENU (n-ethyl-N-nitrosourea) mutagenesis of VC2010, propagated clonally through 10 generations to drive mutations to homozygosity, and subjected to whole-genome sequencing. It is homozygous for a large number of mutations determined from sequence data.	CGC
